# Treatment of Simple Fractures of Distal Aspect of Radius and Ulna in Miniature- and Toy-Breed Dogs with Locking Plate in a Non-Rigid Configuration: An Observational Study of 10 Cases

**DOI:** 10.3390/ani16142162

**Published:** 2026-07-12

**Authors:** Alejandro Blanco, Fidel San Román-Llorens, Fidel San Román, Cristina González, Alberto Climent, Juan José Fuertes, Ana Whyte

**Affiliations:** 1 Servicio de Traumatología, Ortopedia, y Cirugía de la Columna, Hospital de Urgencias Veterinarias de la Región de Murcia, 30110 Murcia, Spain; 2 Department of Animal Pathology, Faculty of Veterinary Science, University of Zaragoza, 50013 Zaragoza, Spain; 3 Hospital Centro Clínico Veterinario de Zaragoza, 50006 Zaragoza, Spain; juanjo7_7@hotmail.com; 4 Department of Animal Medicine and Surgery, Faculty of Veterinary Science, Complutense University of Madrid, 28040 Madrid, Spain; fsanroma@ucm.es

**Keywords:** distal radial fracture, locking plate fixation, veterinary orthopedics, callus formation

## Abstract

Distal radial and ulnar fractures in miniature- and toy-breed dogs are associated with high rates of delayed union, non-union, and refracture. This retrospective observational study evaluated the outcomes of simple distal radial and ulnar fractures treated by open reduction and internal fixation using a locking plate in a non-rigid configuration. Ten fractures in eight dogs weighing less than 5 kg were included. Cases were selected according to predefined biomechanical construct criteria, including a plate bridge ratio > 0.7, plate span ratio > 10, plate working length > 40% of the plate length, and screw density < 0.6. The median radiographic follow-up was 224 days (range, 46–408 days). Radiographic bone healing was achieved in all cases, with a mean time to union of 53 days (range, 28–113 days), and healing occurred faster in skeletally immature dogs. Indirect bone healing with callus formation was observed in 7 of 10 fractures. Minor postoperative complications occurred in three cases and resolved with local treatment. No implant failures or refractures associated with previous screw holes were observed. A reduction in ulnar thickness was identified in evaluable cases, although this finding remained stable during follow-up and did not appear clinically relevant. These findings suggest that locking plate fixation in a non-rigid configuration provides reliable bone healing with a low complication rate in simple distal radial and ulnar fractures of miniature- and toy-breed dogs.

## Simple Summary

Distal radial and ulnar fractures are common in miniature- and toy-breed dogs and are often associated with delayed bone healing, non-union, and refracture after implant removal. These complications have been related to the small size of the bone fragments, the limited blood supply and the high stiffness of some fixation systems. In this study, we evaluated the outcomes of 10 fractures treated with a locking plate applied in a non-rigid configuration designed to reduce construct stiffness while maintaining adequate stability. All fractures achieved radiographic bone healing, and all dogs recovered complete limb function. Bone healing occurred within acceptable time frames, and callus formation was observed in most cases, suggesting indirect bone healing. Only minor postoperative complications were identified, and no implant failures or refractures associated with previous screw holes occurred. A reduction in ulnar thickness was observed during healing in several cases; however, this finding remained stable over time and did not appear to affect limb function. These results suggest that non-rigid locking plate fixation may represent a reliable treatment option for simple distal radial and ulnar fractures in miniature and toy-breed dogs.

## 1. Introduction

Radius and ulna fractures in dogs account for approximately 18% of all bone fractures, with traumatic injury due to a fall being the most common cause [1]. The distal third of the radius and ulna is particularly predisposed to fracture, especially in small-breed dogs [2], and its treatment presents unique challenges in veterinary orthopedics [3,4,5]. Several treatment options have been described, including external coaptation, intramedullary pinning, external skeletal fixation and bone plating; however, casts and intramedullary pinning are associated with high complication rates, including non-union and malunion due to poor mechanical stability and vascular compromise [4,6,7,8]. External skeletal fixation has shown favorable outcomes but requires intensive postoperative management and frequent follow-up [9,10,11,12,13,14,15,16]. Plate osteosynthesis has been widely described for the management of distal radial and ulnar fractures in toy and miniature-breed dogs, using both conventional [17,18,19] and locking systems with open [20,21,22] or minimally invasive [23,24] techniques.

Delayed union, malunion, non-union and refracture after implant removal are common complications of these fractures [25,26,27,28,29,30]. Biomechanical, technical and vascular factors specific to these patients have been proposed as contributors to this condition, although the exact underlying mechanism has not been definitively elucidated.

Initially, it was proposed that internal fixation using plates creates a construct with greater stiffness than the bone itself, resulting in load transfer away from the fracture site toward the implant. This phenomenon, known as stress shielding, leads to cortical osteopenia and bone remodeling, potentially predisposing to delayed union, non-union or refracture after implant removal [31,32,33,34,35]. In addition, the distal radius and ulna in toy breeds are subjected to higher stress concentrations compared with larger dogs, which may further contribute to fracture occurrence and healing complications [2]. Following fracture healing, progressive implant removal (construct dynamization) is commonly performed in orthopedic practice to gradually transfer mechanical loads from the fixation system to the healed bone [36]. This strategy aims to reduce the potential effects of stress shielding and may help limit further bone loss associated with prolonged implant support.

Local anatomical factors, including the small size of bone fragments—particularly the distal segment—the limited contact surface between fracture fragments, the high incidence of short oblique fractures and the distracting forces exerted by the flexor musculature, have historically represented a technical challenge for stable plate fixation in these fractures [7,25,29,30].

The distal fracture region in small-breed dogs has a relatively limited vascular supply [4]. In addition, plate fixation has been shown to negatively affect cortical bone perfusion, and a direct correlation has been demonstrated between the extent of bone resorption and the plate–bone contact area [37,38,39].

The authors hypothesized that the use of a non-rigid locking plate configuration could improve fracture healing in toy-breed dogs by reducing stress shielding, enhancing the preservation and revascularization of the distal radial and ulnar blood supply, and promoting biological bone healing through controlled interfragmentary motion and callus formation while maintaining adequate fracture stability. The aim of this study is to describe the outcomes of simple distal fractures in miniature- and toy-breed dogs treated by open reduction and internal fixation using locking plates with non-rigid configuration and to discuss, in the authors’ opinion, the advantages of this approach in these particular cases.

## 2. Materials and Methods

### 2.1. Inclusion Criteria

**Figure 1:**
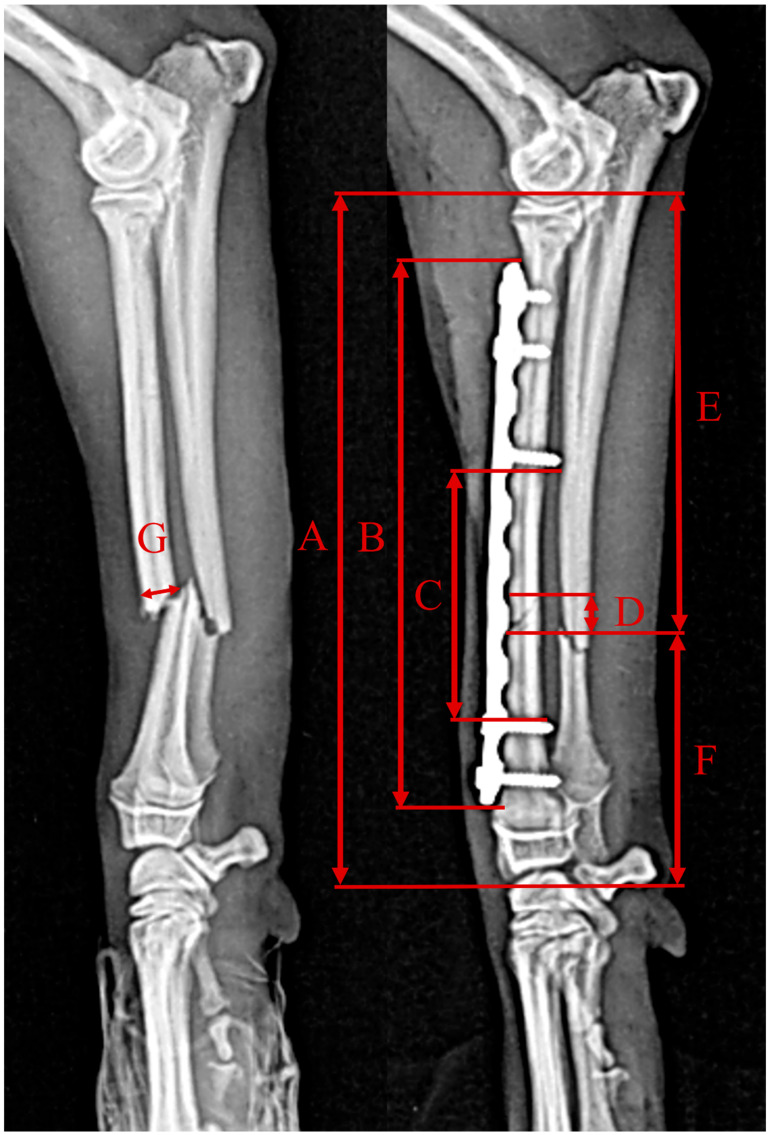
Preoperative and immediate postoperative mediolateral radiographic projections in case 4 showing how the measurements of each case were performed to calculate the biomechanical construct configuration. Total radial length (A), plate length (B), distance between the two screws closest to the fracture (C), fracture length (D), proximal segment length (E), distal segment length (F) and radial width at its narrowest point (G).

A retrospective study was conducted at the Hospital de Urgencias Veterinarias de la Región de Murcia, including cases of fractures of the distal aspect of the radius and ulna fractures in miniature- and toy-breed dogs (<5 kg) treated between January 2023 and February 2026. The inclusion criteria for this study were:

Fracture type: closed, transverse or short oblique fractures affecting the distal aspect of the radial diaphysis. To confirm this criterion, measurements (Figure 1) were performed using RadiAnt DICOM Viewer 2025.2 (Medixant, Poznań, Poland) on the mediolateral radiographic projection in each case. The total radial length (A) was measured postoperatively from the most proximal aspect of the radial head to the most distal aspect of the distal radial epiphysis, and the distal fragment length (F) was measured from the fracture line to the most distal aspect of the radial epiphysis. Only cases with an F/A ratio ≤ 0.4 were included. In these cases, the following measurements were also recorded: total plate length (B), distance between the proximal and distal screws adjacent to the fracture (C) and fracture length (D). Additionally, the craniocaudal radial width at its narrowest point (G) was measured in mediolateral preoperative projections.

Surgical treatment: Fractures treated by open reduction and internal fixation using a single titanium cut-to-length locking reconstruction plate (Healthtech Solutions, Munich, Germany) placed on the cranial aspect of the radius, without the use of any additional fixation or external coaptation.

Plate and screw construct: Only cases meeting the following measurements, as previously described [24], were included: plate bridge ratio (PBR) > 0.7, which relates total plate length to total radial length (B/A); plate span ratio (PSR) > 10, relating total plate length to fracture length (B/D); plate working length (PWL) > 40% of the total plate length, defined as the distance between the screws closest to each side of the fracture site; and screw density (SD) < 0.6, defined as the number of screws divided by the total number of plate holes. Additionally, the ratio between plate thickness and the craniocaudal radial width at its narrowest point did not exceed 0.6.

Follow-up: Only cases with sufficient clinical and radiographic records to allow longitudinal follow-up until bone healing or non-union were included.

### 2.2. Data Collection

For each case, data on breed, age, sex and body weight were recorded. Patients were classified as skeletally immature or mature based on age and the presence of open growth plates on radiographic evaluation. Fractures were classified as acute (<10 days from injury) or chronic (>10 days from injury).

The day of fracture treatment was recorded as day 0. Fracture reduction was assessed on mediolateral and craniocaudal radiographic projections. Reduction was classified according to the 50/50 rule as acceptable when at least 50% cortical apposition and contact between fragments were present in both projections or inadequate otherwise.

Regarding construct configuration, the screw diameter, the screw position within the plate, and whether screws were monocortical or bicortical were recorded. In addition, the total number of plate holes was documented.

**Figure 2:**
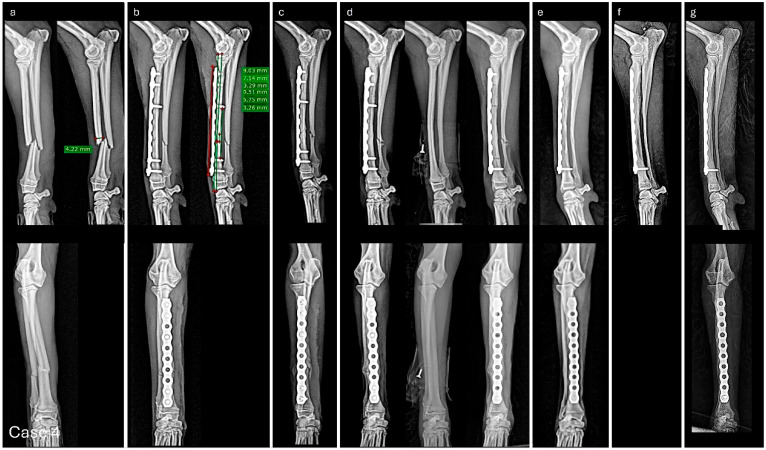
Radiographic follow-up of case 4. Preoperative mediolateral and craniocaudal projections showing measurement of radial thickness at its narrowest point (**a**). Immediate postoperative projections showing plating configuration and measurements (**b**). At day 9 (**c**), bone remodeling and initial callus formation were observed. At day 30 (**d**), radiographic bone healing of the radius was completed, and the ulna showed reduced thickness compared to the contralateral limb; screws 2, 3, 4 and 5 were removed. At day 79 (**e**), screw holes were filled with bone tissue and screw 1 was removed (**f**). Radiographic appearance at day 408 (**g**).

All radiographic studies of the affected limb and the contralateral healthy limb obtained during the follow-up period were reviewed. These studies were used to determine whether the ulnar thickness at its distal aspect was reduced at the time of bone healing and whether this reduction remained stable at the last radiographic follow-up (Figure 2). Ulnar thickness reduction was assessed quantitatively by measuring the distal ulnar thickness of the affected limb at the time of bone healing and comparing it with the corresponding measurement obtained from the contralateral healthy limb at the same time point. All measurements were performed on mediolateral radiographic projections using RadiAnt DICOM Viewer (Medixant, Poznań, Poland).

### 2.3. Follow-Up

The follow-up time was calculated from day 0 to the last radiographic record obtained at the same center where the surgical treatment was performed and was classified according to standard time frames as perioperative (0–3 months), short-term (3–6 months), mid-term (6–12 months) or long-term (>12 months). Complications were recorded throughout the follow-up period and classified as catastrophic, major or minor [40]. Catastrophic complications were defined as those resulting in permanent unacceptable function, directly related to death, or leading to euthanasia. Major complications were defined as those requiring additional treatment to resolve, including either surgical intervention or medical management according to the current standard of care. Minor complications were defined as those not requiring additional surgical or medical treatment for resolution.

The time to radiographic bone healing was determined and defined as restoration of the cortical continuity of the radius in orthogonal projections. Bone healing was classified as secondary when callus formation was observed at any point during follow-up or as primary (direct) when no callus formation was detected. At that time, radiographic comparison in mediolateral projection of the affected ulnar thickness with the contralateral limb was performed. Functional limb use at the time of bone healing was also recorded and classified as complete, acceptable or unacceptable [40]. Implant-related complications (including implant failure, loosening or infection) were recorded, as well as whether or not implant removal was performed after bone healing (partial or complete).

## 3. Results

**Figure 3:**
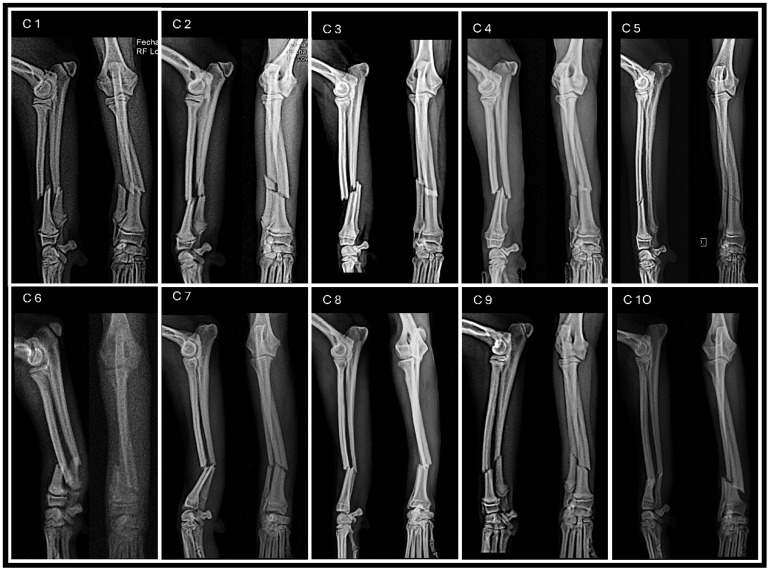
Mediolateral and craniocaudal radiographic projections obtained at the time of presentation in the 10 cases included in the study. Each case is identified by its corresponding number preceded by the letter C in the upper left corner of each image.

A total of 10 acute fractures met the inclusion criteria (Table 1), corresponding to 8 patients, as two dogs sustained bilateral fractures at different time points (cases 1 and 2; cases 4 and 8). At the time of fracture presentation, the most common breed was Pomeranian (*n* = 4), followed by Yorkshire Terrier and Miniature Poodle (*n* = 3 each). There were 6 males and 4 females included in the study. The mean body weight was 3.12 kg (range, 0.9–4.8 kg) and the mean craniocaudal radial width at its narrowest point was 0.35 cm (range, 0.24–0.46 cm). The mean age was 6.6 months (range, 3–12 months); 7 fractures occurred in skeletally immature dogs and 3 in skeletally mature dogs. All radial fractures showed a simple short oblique configuration (Figure 3) extending from proximolateral to distomedial. This pattern was similar in the ulnar fractures, except in case 2, which presented a complex ulnar fracture.

**Figure 4:**
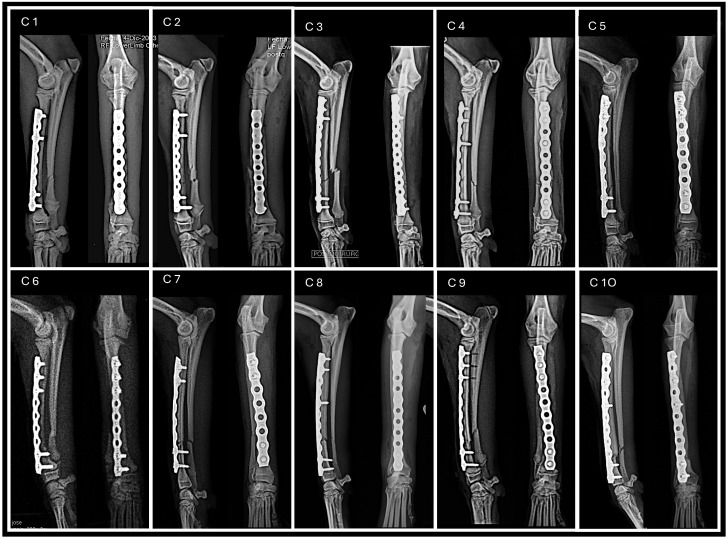
Mediolateral and craniocaudal radiographic projections showing fixation using a single locking plate in each of the 10 cases included in the study. Each case is identified by its corresponding number preceded by the letter C in the upper left corner of each image.

**Figure 5:**
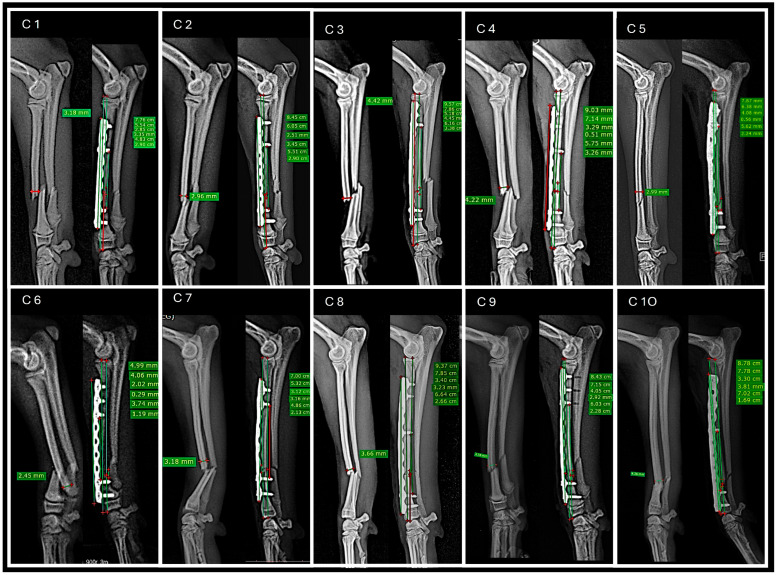
Measurements performed on the preoperative and postoperative mediolateral projections in the 10 cases included in the study. Each case is identified by its corresponding number preceded by the letter C in the upper left corner of each image.

Fracture reduction was classified as acceptable in all cases (Figure 4). The construct configuration parameters (Figure 5) showed a mean PBR of 0.81 (range, 0.71–0.89), mean PSR of 20.5 (range, 11–37), mean screw density of 0.47 (range, 0.40–0.60) and mean WL of 3.49 cm (range, 2.02–5.18 cm), corresponding to a mean of 56% (range, 44–69%) of the total plate length. The mean number of plate holes was 10.0 (range, 8–13). The mean number of empty plate holes between the screws closest to each side to the fracture was 4.73 (range, 4–8). The screw diameter was 1.5 mm in one case (case 6) and 2.0 mm in nine cases. Distal fixation was achieved using two bicortical screws in all cases. Proximal fixation consisted of two bicortical screws in four cases, and three screws in different mono- or bicortical configurations in six cases (the results of the construct configuration are presented in Supplementary Material Table S1).

The median radiographic follow-up time was 224 days (range, 46–408 days), being classified as perioperative in 1 case, short-term in 4 cases, mid-term in 3 cases and long-term in 2 cases. The radiographic follow-up for all cases is provided in Supplementary Materials.

**Figure 6:**
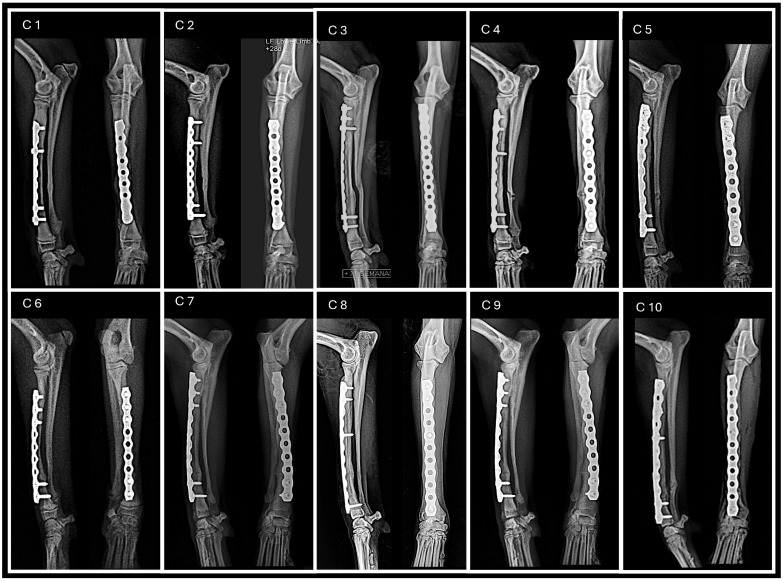
Mediolateral and craniocaudal radiographic projections showing radiographic bone healing in the 10 cases included in the study. Each case is identified by its corresponding number preceded by the letter C in the upper left corner of each image.

**Figure 7:**
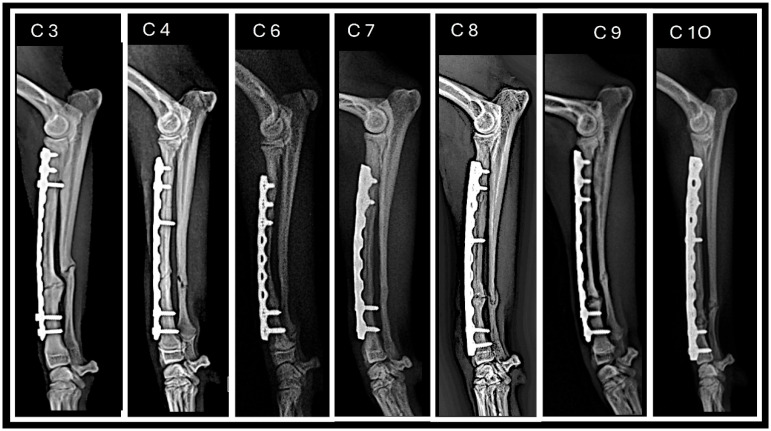
Callus formation is shown in the 7 cases, in which indirect bone healing could be confirmed. Each case is identified by its corresponding number preceded by the letter C in the upper left corner of each image.

**Figure 8:**
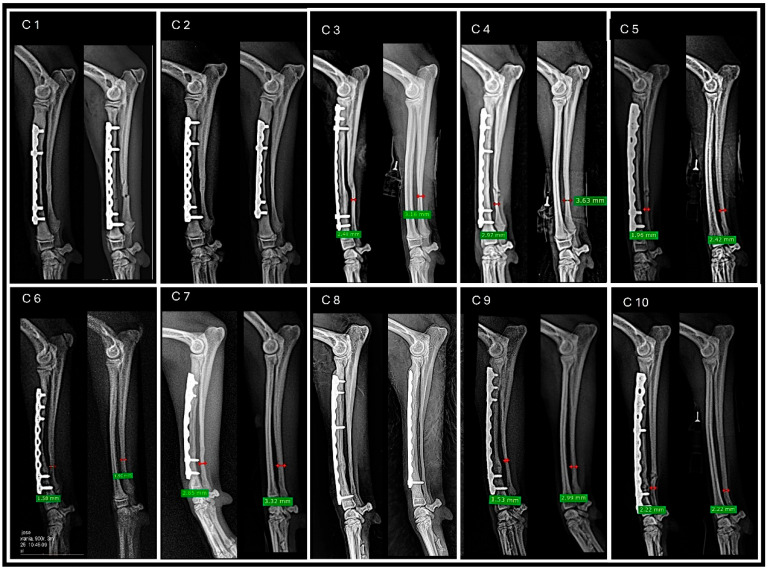
Mediolateral radiographic projections of the affected limb (left image) and the contralateral limb (right image) obtained at the time of bone healing. The figure illustrates the comparison of ulnar thickness measurements between the affected and contralateral limbs in each case included in the study. Quantitative comparison was not possible in cases C1, C2 and C8 because the contralateral limb had sustained a previous fracture before the time of bone healing assessment.

Radiographic bone healing (Figure 6) was achieved in all cases, with a mean time to union of 53 days (range, 28–113 days). The healing time was shorter in skeletally immature dogs (47.9 days; range, 28–113 days) compared to skeletally mature dogs (66 days; range, 56–76 days). Callus formation (Figure 7) was observed in 7 out of 10 cases (70%) during the healing process. At the time of bone healing, in 7 cases (cases 3, 4, 5, 6, 7, 9 and 10), it was possible to compare ulnar thickness (Figure 8) on the mediolateral projection of the affected limb with that of the contralateral healthy limb. A reduction in ulnar thickness was observed in all of these cases except case 10. In the remaining three cases (cases 1, 2 and 8), objective comparison was not possible due to previous fractures affecting the contralateral limb; however, subjective evaluation suggested the presence of ulnar thinning. Limb function was classified as complete in all cases, with full use of the affected limb without pharmacological support.

Three minor postoperative complications were recorded, consisting of seroma formation in two cases and incisional complications in one case, all of which resolved with local treatment. In two cases, focal osteolysis of the ulna was observed at the point of contact with the tip of a proximal screw, which was resolved after implant removal.

In nine cases with short-term or longer follow-up, implant removal was performed, either totally (*n* = 4) or partially (*n* = 5). Removal was progressive in 7 cases (cases 1, 2, 4, 5, 6, 7 and 9) and performed in a single stage in two cases (cases 3 and 8). In one case (case 10), no implant removal was done at the time of the study.

**Figure 9:**
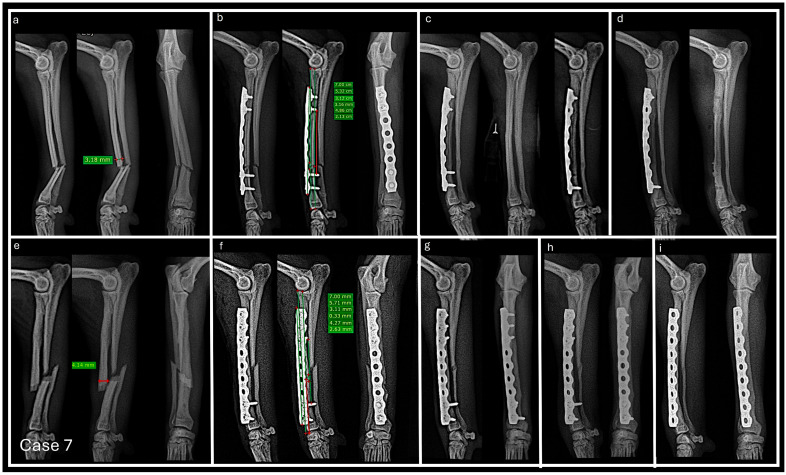
Radiographic follow-up of case 7. Preoperative mediolateral and craniocaudal projections showing measurement of radial thickness at its narrowest point (**a**). Immediate postoperative projections showing plating configuration and measurements (**b**). At 66 days, bone healing is observed, with thinning of the ulna compared to the contralateral limb and screws 2 and 3 were removed (**c**). At 111 days, no further ulnar thinning was observed and the remaining implants were removed (**d**). Seventy days later, a second fracture of the radius and ulna occurred in a more proximal location relative to the initial fracture (**e**), which was treated with a locking plate in a non-rigid configuration again (**f**). Follow-up at 35 days (**g**), showing bone healing in progress, and 70 days (**h**), showing complete bone healing. At day 247 (**i**), the final follow-up is shown; the screws had been progressively removed, the screw holes were filled with bone tissue and the ulnar thickness remained stable.

In one case (case 7) (Figure 9), a second fracture occurred 70 days after implant removal following a fall from a chair. Radiographic measurements confirmed that the new fracture site did not coincide with the first fracture location or with any previous screw holes; therefore, it was not classified as a refracture or implant-related complication. The fracture was treated using a 2.0 mm locking reconstruction plate with nine holes, applying three proximal and two distal bicortical screws, leaving four empty holes between the screws closest to the fracture site. Radiographic bone healing was achieved at 70 days and the fixation was subsequently dynamized by the removal of all screws.

**Figure 10:**
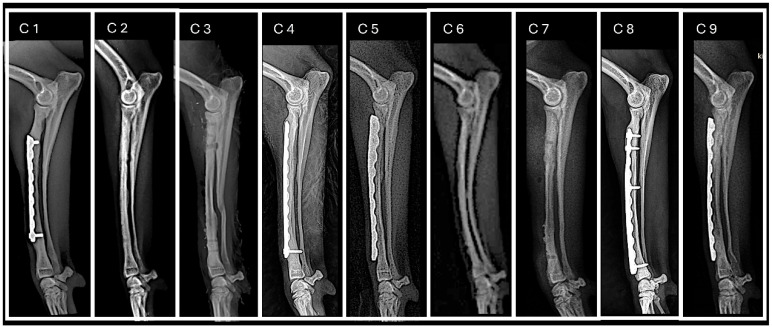
Mediolateral radiographic projections obtained at the final radiographic follow-up of the nine cases included in the study with follow-up extending beyond the perioperative period. Case 10 was excluded because only perioperative radiographs were available. Each case is identified by its corresponding number, preceded by the letter C, in the upper left corner of each image.

At the end of the follow-up period, all cases showed complete functional use of the affected limbs, and in all cases with short-term or longer radiographic follow-up, the ulnar thickness remained stable compared to the previous evaluation (Figure 10).

## 4. Discussion

Successful treatment of simple distal radial fractures in miniature and toy dogs using locking plates has previously been reported [20,21,22], characterized by low PBR and PSR values and high screw density, which can be considered stiff configurations. An exception is represented by reports using minimally invasive plate osteosynthesis (MIPO) techniques, in which the preservation of periosteal vascularization and the fracture hematoma is emphasized as the main advantage [23,24]. This study reports excellent outcomes using open reduction and internal fixation with a locking plate in a non-rigid configuration. A thorough discussion of these findings is warranted.

In locking plate fracture fixation, multiple variables can be modified, including the plate length, number and type of screws, screw distribution and fracture gap size. These factors directly influence implant stresses, construct stiffness, interfragmentary strain and ultimately the biological environment for fracture healing [41,42,43,44]. Traditionally, excessive construct stiffness has been associated with stress shielding [31,32,34]. This concept remains controversial, as bone resorption beneath plates has been observed regardless of plate stiffness [45], and because similar construct stiffness has been demonstrated in toy- and large-breed dogs despite the absence of bone resorption in the latter [46]. In the present study, only constructs fulfilling predefined criteria for non-rigid fixation [42,47,48] in terms of PBR, PSR, working length and screw density were included. We believe that this configuration is advantageous not only for reducing the potential effects of stress shielding, but more importantly for promoting callus formation. Secondary bone healing, characterized by callus formation, has been associated with stronger union compared to primary bone healing [49], which typically occurs under conditions of high construct stiffness. Bone healing through callus formation was observed in all cases except three. In these cases, the first follow-up radiographs were obtained when the healing process was already complete, and prior callus formation may have undergone remodeling and therefore not been detectable. The retrospective and observational nature of the study limits the ability to draw definitive conclusions in this regard, although the findings still support the proposed hypothesis.

Locking plate fixation has been shown to better preserve periosteal vascularization at the fracture site by minimizing the area of contact [39,47,50,51] and reducing pressure between the plate and the bone surface. However, this has been questioned by both experimental and clinical studies [37,52,53], which suggest that the actual plate–bone contact area is influenced more by the complex surface morphology of the bone and the surgeon’s ability to accurately contour the plate than by the plate design itself. The authors believe that the construct configuration used in the present study minimizes plate–bone contact and pressure specifically within the working length, where the absence of screws reduces the degree of plate apposition to the underlying bone surface. This region is also considered to have a more compromised vascular supply [4], making this effect potentially clinically relevant.

The use of a titanium plate with a long working length improves resistance to fatigue failure [54]. Nevertheless, accurate fracture reduction without a residual gap between bone segments is still recommended so that controlled interfragmentary motion can occur while maintaining adequate construct stability. Additionally, the use of long plates spanning a greater portion of the radius places the proximal end of the construct closer to the proximal radial epiphysis, reducing the lever arm generated at this location and therefore potentially decreasing the risk of stress concentration fractures [55].

The reduction in ulnar thickness observed in all cases may be related to the initial trauma, the surgical treatment or a combination of both factors. The authors believe that implant removal, whether performed progressively or in a single stage, may contribute to halting this process. However, in case 8, in which only one screw was removed, the ulnar thickness remained stable at the end of the radiographic follow-up, 252 days after treatment, suggesting that this phenomenon may be self-limiting and not solely dependent on implant removal. The dynamization process consisted of a progressive and controlled implant removal protocol performed under sequential radiographic monitoring. Implant removal was carried out gradually, with periodic radiographic evaluations to assess fracture stability, bone remodeling, and changes in ulnar thickness. Dynamization was discontinued when the observer determined that the reduction in ulnar thickness had stabilized and no further progression was evident. Depending on the radiographic findings, the process could conclude either with complete implant removal or with retention of part of the fixation construct. In cases in which indirect bone healing with abundant callus formation was observed, such as case 3, implant removal was preferentially performed in a single stage. In the authors’ opinion, the presence of a robust callus provided sufficient mechanical support to minimize the risk of refracture, making progressive dynamization unnecessary and avoiding additional anesthetic procedures.

No refractures were observed following implant removal, suggesting that bone healing was mechanically robust. The authors believe that screw placement confined to the proximal and distal regions of the radius—areas subjected to lower mechanical stress in these patients—may reduce the risk of refracture through previous screw holes.

This study has several limitations, including a relatively small sample size, non-standardized radiographic follow-up timing, its retrospective nature and the absence of a control group, which limits direct comparison with other fixation strategies. Despite the relatively small sample size, the homogeneity of the individuals included and the standardized intervention performed in all cases provide important strength to the study.

## 5. Conclusions

In conclusion, the use of non-rigid locking plate constructs (plate bridge ratio (PBR) > 0.7; plate span ratio (PSR) > 10; working length (PWL) > 40% of the plate length; screw density < 0.6) may be considered a potential treatment approach for simple distal radial fractures in miniature- and toy-breed dogs (body weight < 5 kg; craniocaudal radial width < 0.46 cm). In the population of animals included in the present study, this configuration provided reliable bone healing within acceptable time frames and was associated with a low rate of complications across all follow-up periods. The construct design, defined by reduced stiffness, may promote callus formation and the preservation of vascular supply. Although a reduction in ulnar thickness was observed, this finding remained stable over time and did not appear to have clinical relevance. Further prospective studies with larger sample sizes and control groups are warranted to confirm these findings.

## Figures and Tables

**Table 1:** Data collection for all cases included in the study. Abbreviations: f, female; I, skeletally immature; M, skeletally mature; m, male; ms, months. * Percentage of thinning of the affected ulna compared with the healthy contralateral ulna.

Case	Signalment					Plating Configuration				Follow-Up	Bone Healing			Complications	Implant Removal
	Breed	Sex	Age (ms)	Weight (kg)	Skeletal Maturity	Plate Bridge Ratio	Plate Span Ratio	Screw Density	Working Length (% of Total Plate Length)	Days Since Treatment to Last Radiographic Examination	Days Since Treatment to Bone Healing	Callus Formation	Ulnar Thinning at the Time of Radial Bone Healing (%) *		
1	Yorkshire Terrier	f	3	2.5	I	0.71	15	0.4	51	153	30	No	No control group	No	Partial
2	Yorkshire Terrier	f	4	3	I	0.77	26	0.4	53	120	28	No	No control group	Focal osteolysis of the ulna at the point of contact with screw 2	Complete
3	Miniature Poodle	m	8	4.8	I	0.82	17	0.4	66	119	113	Yes	Yes (11%)	Focal osteolysis of the ulna at the point of contact with screw 3	Complete
4	Miniature Poodle	m	5	3.5	I	0.83	37	0.5	44	408	30	Yes	Yes (8%)	Seroma	Partial
5	Pomeranian	m	6	3.2	I	0.81	11	0.4	64	332	30	No	Yes (8%)	No	Partial
6	Pomeranian	f	3	0.9	I	0.81	14	0.6	50	257	28	Yes	Yes (8%)	No	Complete
7	Pomeranian	m	8	2.6	M	0.76	17	0.5	59	251	66	No	Yes (25%)	No	Complete
8	Miniature Poodle	m	11	4	M	0.84	24	0.5	43	252	56	Yes	No control group	Seroma	Partial
9	Yorkshire Terrier	f	6	3.7	I	0.84	24	0.5	57	135	76	Yes	Yes (51%)	Incisional dehiscence	Partial
10	Pomeranian	m	12	3.5	M	0.89	20	0.5	48	76	76	Yes	No	No	Perioperative follow-up

## Data Availability

The original contributions presented in this study are included in the article/Supplementary Materials. Further inquiries can be directed to the corresponding author.

## Supplementary Materials

The following supporting information can be downloaded at: https://www.mdpi.com/article/10.3390/ani16142162/s1. Table S1: Abbreviations and notes: bi, bicortical screws; mo, monocortical screws.
