# Supplementary material for: Treatment of Simple Fractures of Distal Aspect of Radius and Ulna in Miniature- and Toy-Breed Dogs with Locking Plate in a Non-Rigid Configuration: An Observational Study of 10 Cases
Source: Animals (Basel). 2026 Jul 12;16(14):2162. doi: 10.3390/ani16142162 (PMC13405760; doi:10.3390/ani16142162)
Supplement: Supplementary file 1 [file animals-16-02162-s001.zip › Table S1.pdf]

Case	Plating configuration								
	Plate bridge ratio	Plate spam ratio	Screw density	Working Length (% of total plate length)	Number of holes	Number of screws	Screw position in plate	Proximal screws number and type	Distal Screws Number and type
**1**	0.71	15	0.4	51	9	4	1-3-8-9	2; bi-bi	2; bi-bi
**2**	0.77	26	0.4	53	10	4	1-3-9-10	2; bi-bi	2; bi-bi
**3**	0.82	17	0.4	66	13	5	1-2-3-12-13	3; mo-mo-bi	2; bi-bi
**4**	0.83	37	0.5	44	10	5	1-2-4-9-10	3; mo-mo-bi	2; bi-bi
**5**	0.81	11	0.4	64	9	4	1-2-8-9	2; bi-bi	2; bi-bi
**6**	0.81	14	0.6	50	9	5	1-2-3-8-9	3; mo-mo-mo	2; bi-bi
**7**	0.76	17	0.5	59	8	4	1-2-7-8	2; bi-bi	2; bi-bi
**8**	0.84	24	0.5	43	11	5	1-2-4-10-11	3; bi-bi-bi	2; bi-bi
**9**	0.84	24	0.5	57	10	5	1-2-3-9-10	3; bi-bi-bi	2; bi-bi
**10**	0.89	20	0.5	48	11	5	1-3-5-10-11	3; mo-mo-bi	2; bi-bi

Table S1. Abbreviations and notes: bi, bicortical screws; mo, monocortical screws. *Screw position within the plate was described according to the hole occupied by each screw, considering hole 1 as the most proximal hole of the plate
