# Supplementary figures and images for: Treatment of Simple Fractures of Distal Aspect of Radius and Ulna in Miniature- and Toy-Breed Dogs with Locking Plate in a Non-Rigid Configuration: An Observational Study of 10 Cases
Source: Animals (Basel). 2026 Jul 12;16(14):2162. doi: 10.3390/ani16142162 (PMC13405760; doi:10.3390/ani16142162)

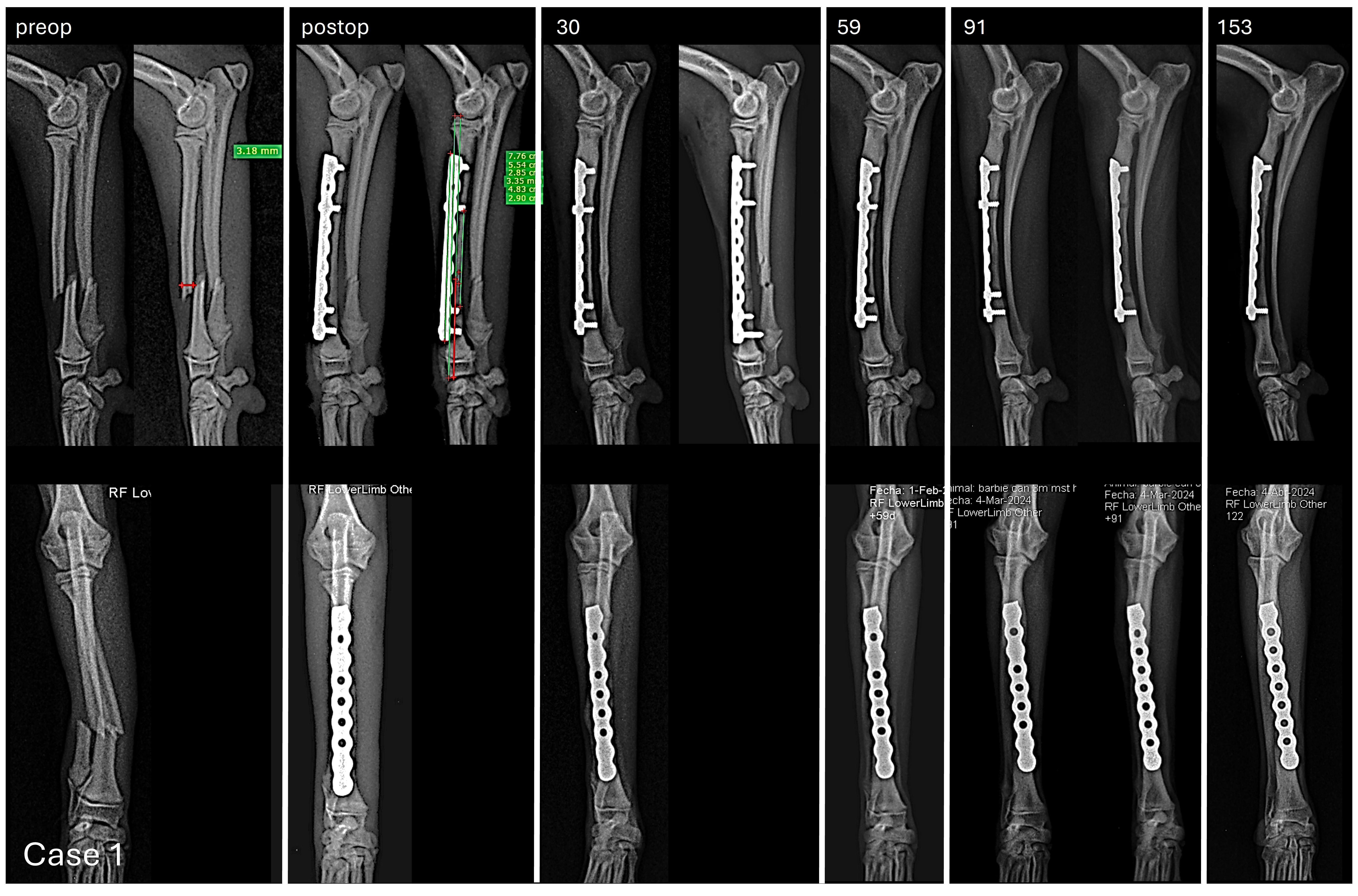

Supplement: Supplementary file 1 [file animals-16-02162-s001.zip › Case 1.jpg]

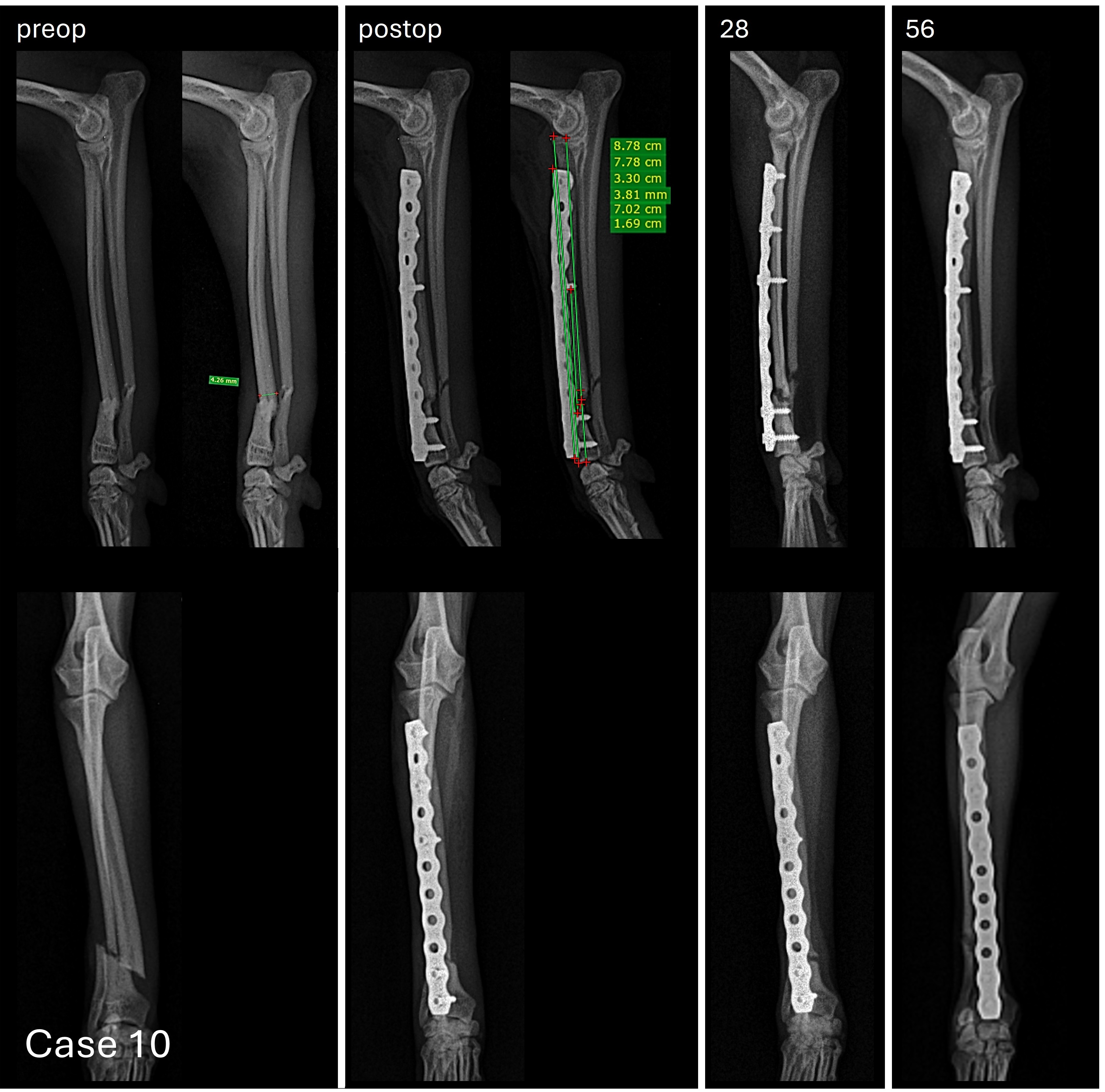

Supplement: Supplementary file 1 [file animals-16-02162-s001.zip › Case 10.jpg]

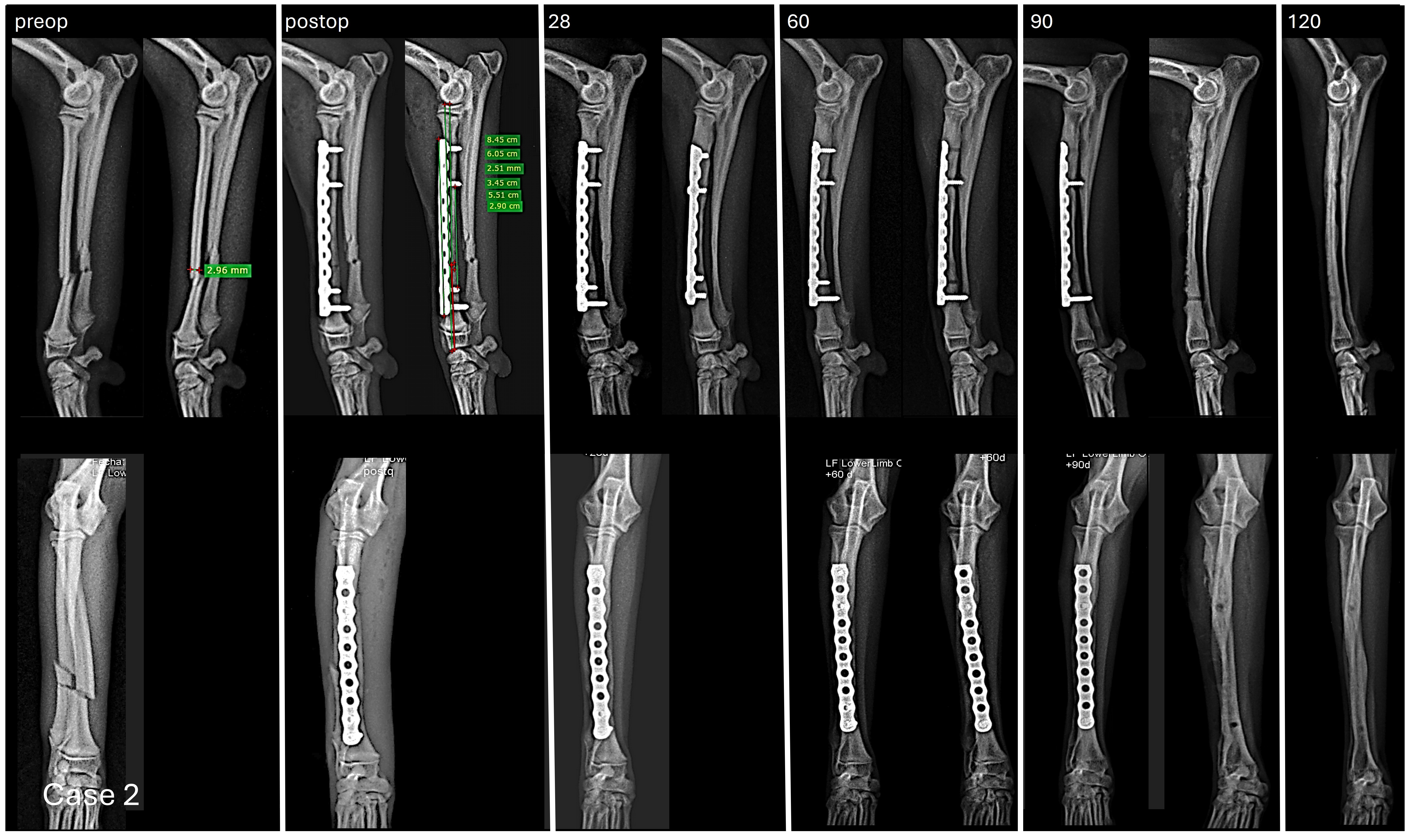

Supplement: Supplementary file 1 [file animals-16-02162-s001.zip › Case 2.jpg]

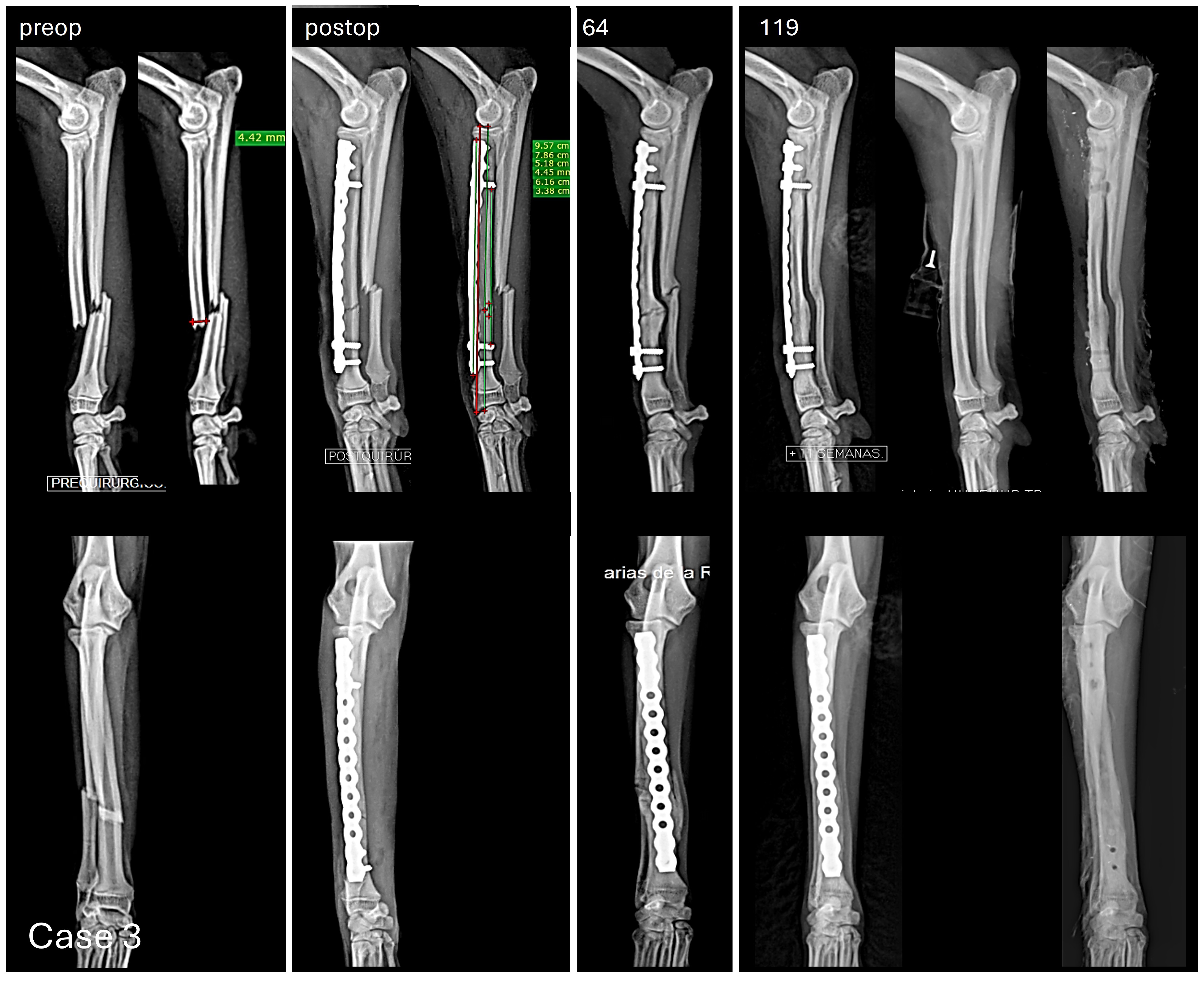

Supplement: Supplementary file 1 [file animals-16-02162-s001.zip › Case 3.jpg]

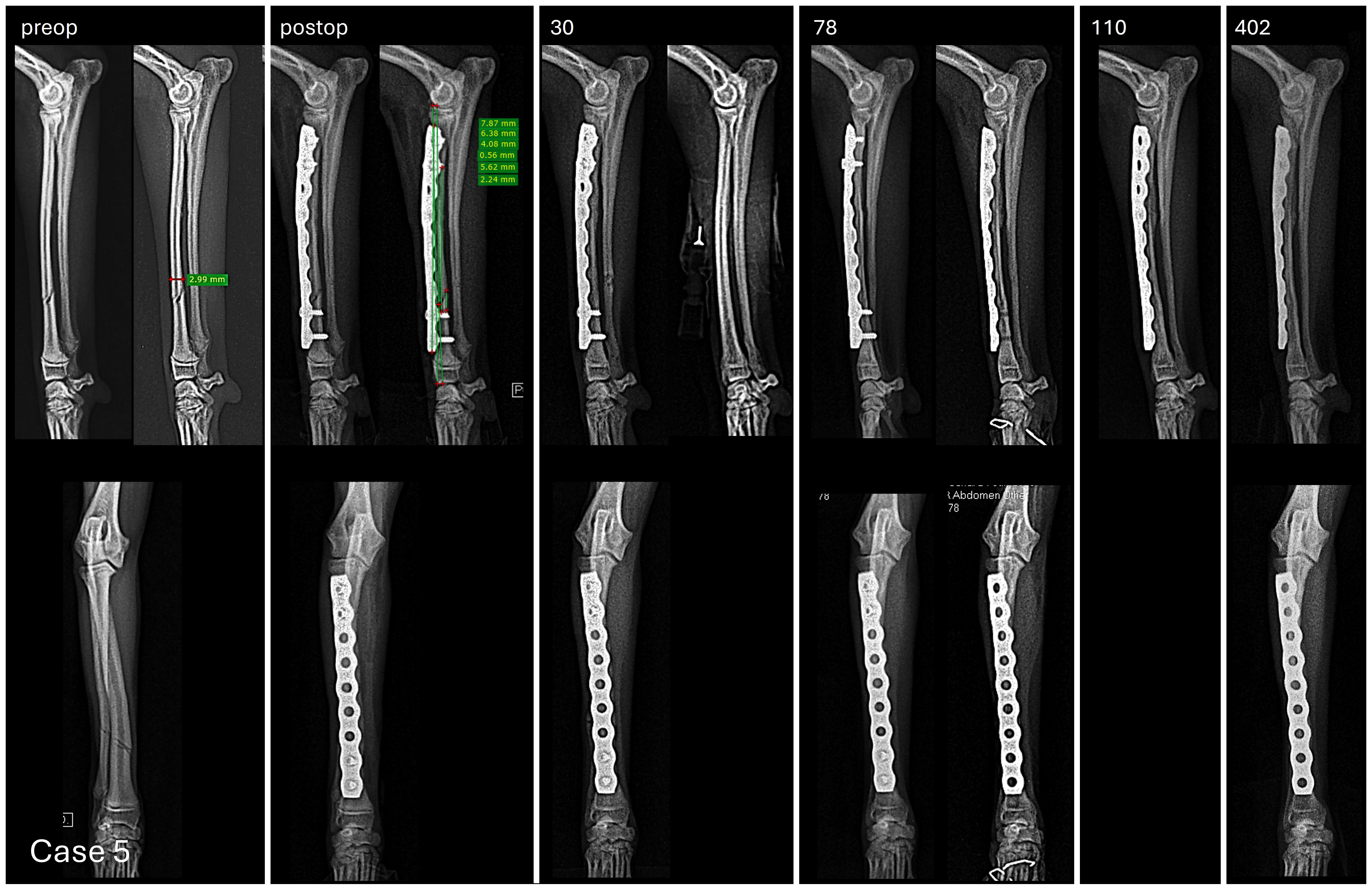

Supplement: Supplementary file 1 [file animals-16-02162-s001.zip › Case 5.jpg]

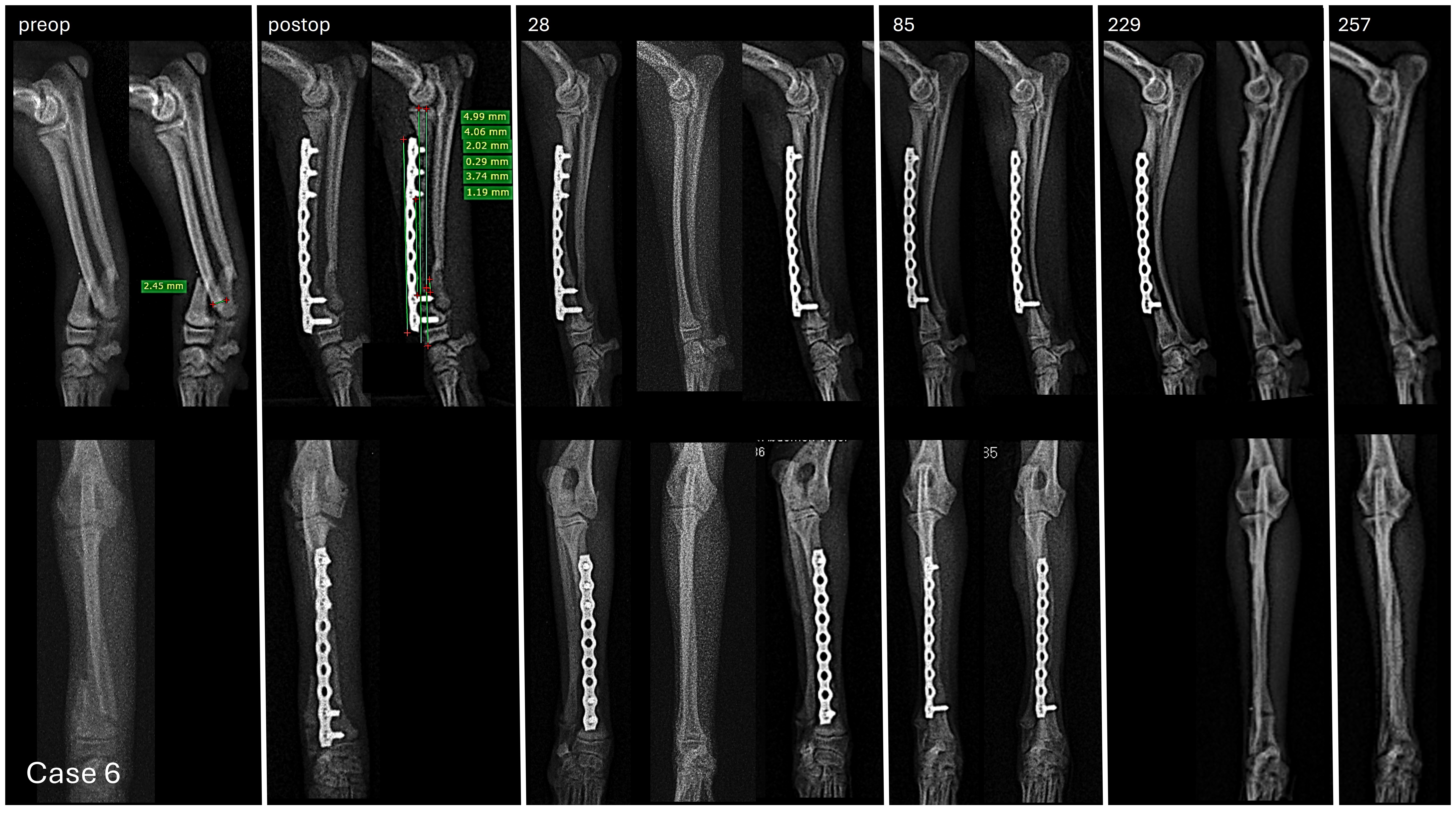

Supplement: Supplementary file 1 [file animals-16-02162-s001.zip › Case 6.jpg]

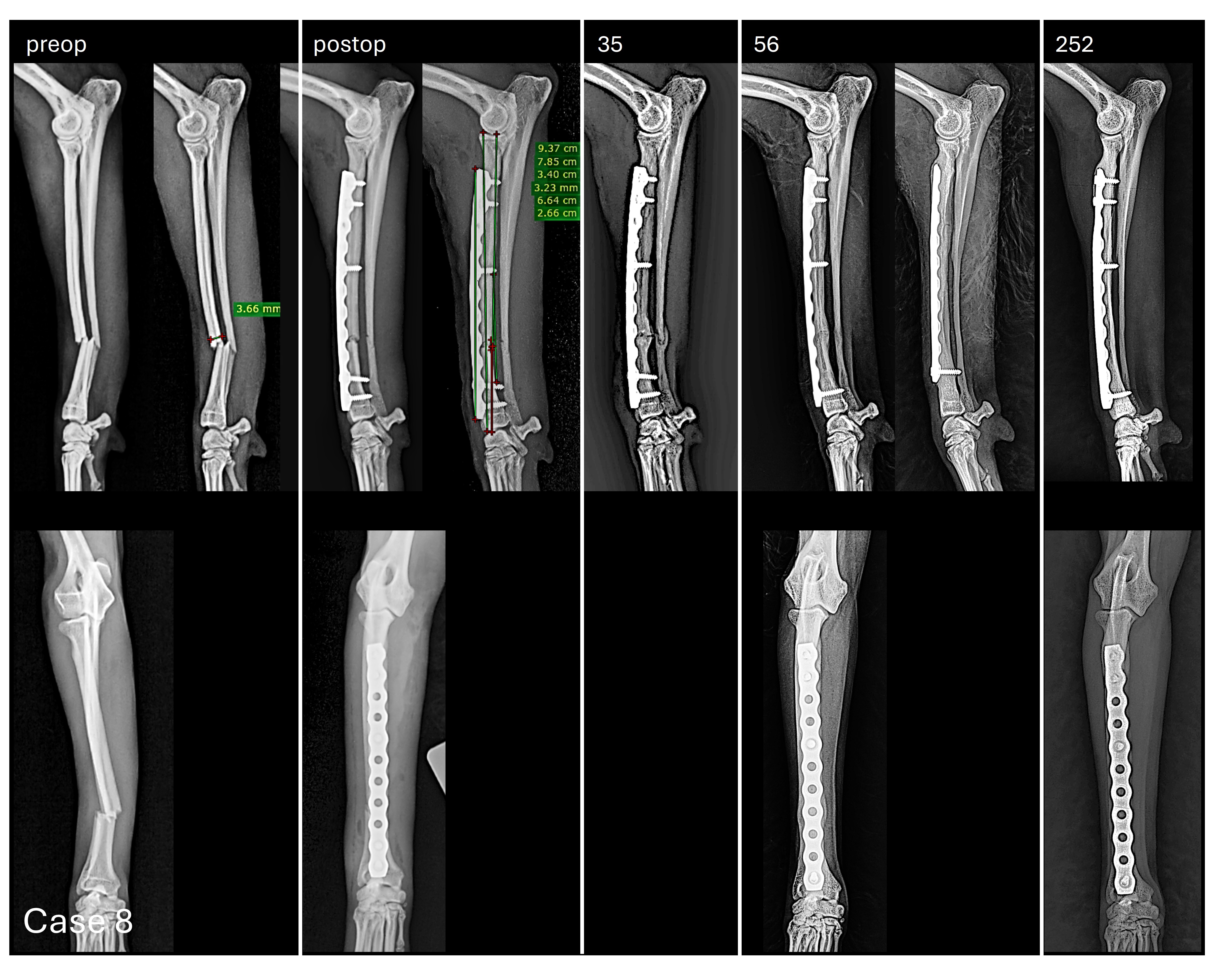

Supplement: Supplementary file 1 [file animals-16-02162-s001.zip › Case 8.jpg]

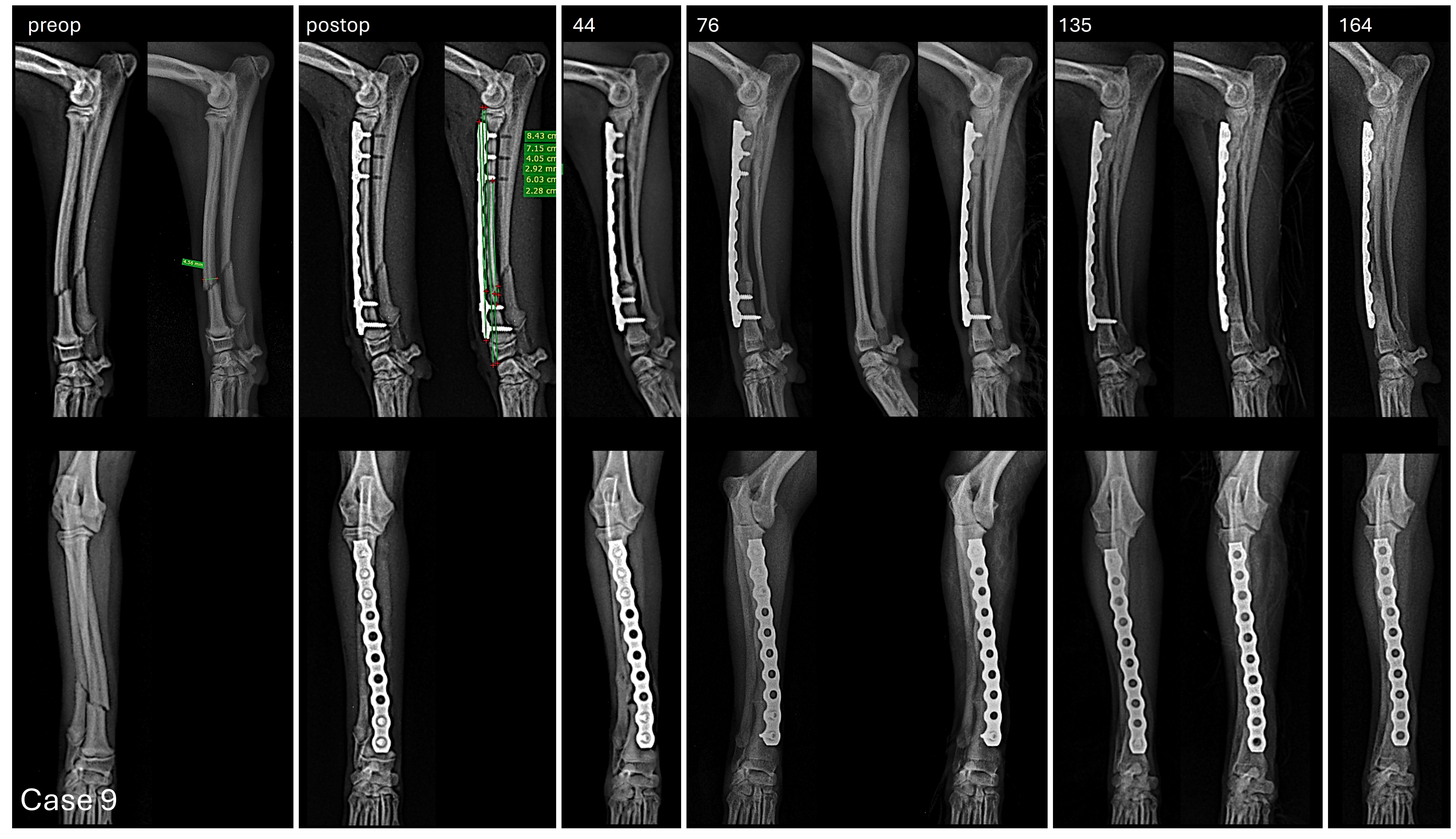

Supplement: Supplementary file 1 [file animals-16-02162-s001.zip › case 9.jpg]
